# Surgical field fire and skin burns caused by alcohol-based skin preparation

**DOI:** 10.4103/0974-2700.66530

**Published:** 2010

**Authors:** Rajan Patel, K D Chavda, Santosh Hukkeri

**Affiliations:** Department of Surgery, NHL municipal medical college, Ahmedabad, India

Sir,

Cases of fire in the operating room are reported in the literature. The factors that may initiate these fires include alcohol-based surgical prep solutions, electrosurgical equipment, flammable drapes, etc. We report one such case of fire in the surgical field, which was caused while the removal of open end of packed bandage in a patient of severe liver trauma. This letter is to highlight an additional risk factor for surgical field fire.

Our patient was a 25 year- old male admitted with severe blunt abdominal trauma 1 hour before. On examination, the patient’s pulse rate 124/minute, blood pressure was 70/50 mm Hg and he had severe pallor. Urgent ultrasonography suggested severe liver injury with massive hemoperitoneum. The patient was immediately shifted to the operation theatre. Our common practice for is to use alcohol (70% w/v) based skin preparation and care for complete drying of skin and then cover drapes. Emergency exploratory laparotomy revealed severe liver parenchymal injury in segments V, VI and VII with active bleeding (Grade IV). Liver packing was carried out with five large-sized cotton bandages tied to each other end to end, and packed around lateral, anterior, and superior surfaces of liver, and three more similar bandages packed around the inferior surface and around the gallbladder. Tails of both the bandages were taken out with two separate abdominal incisions. Further treatment and blood component transfusions had prevented coagulopathy and the patient became hemodynamically stable.

**Figure 1:**
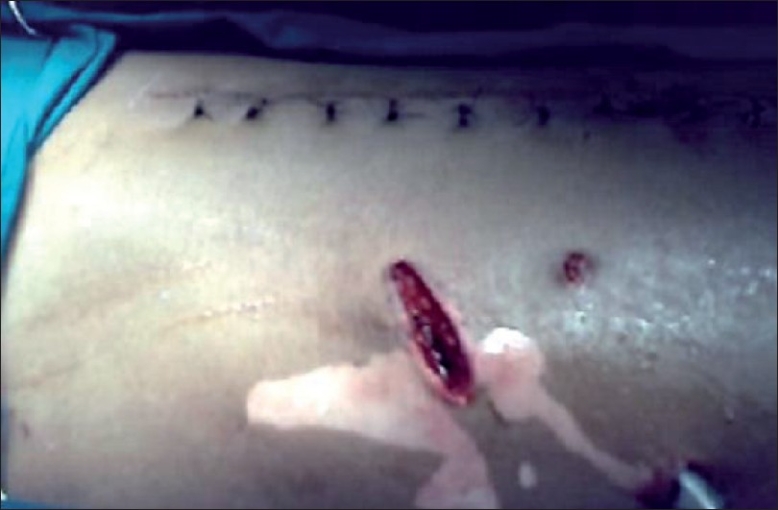
Intraoperative photo showing laparotomy wound with explored superior packed wound surrounded by first-degree burns, other tail site, and drain

After 48 hours of observation, the patient was taken in operation theatre for pack removal under local anesthesia, with preparation to do laparotomy if required. The patient’s abdominal skin was painted with ethyl alcohol 70% w/v. We waited for 3 minutes for evaporation of alcohol and wiped the skin for any retaining alcohol. Then 20ml of 1% Lignocaine was infiltrated around the packed upper tail, and skin incision was made on both sides of the tail to expand local visibility. Electrocautery for hemostasis was just started and accidental fire took place around the incision. The electrocautery was switched off and the fire was rapidly extinguished within seconds with saline and packing with wet gauges. The patient suffered first-degree epidermal burns around the area [Figure 1]. The reason for fire was discovered to be from burned tail of packed bandage, which had retained alcohol. Packed bandages were removed under observation and there was no further bleeding from liver. The first-degree burns [Figure 1] healed with Fremycetin tulle dressing. The patient was discharged on ninth post-operative day.

Operating room fires are uncommon. Nearly 70% of these fires are related to use of electro-surgical equipment and, in 72% of cases, an oxygen-enriched atmosphere has been shown to have contributed to the fire. It has also been noticed that there is a significant risk of fire when alcohol-based surgical prep solutions are used for skin preparation.[1] The fire triangle describes three elements necessary for initiation of a fire, i.e. heat, fuel, and an oxidizer. In the case of operating room fires, an electrosurgical diathermy unit most often provides heat to ignite the flammable substance, although lasers and fiberoptic light sources can be potent heat sources. The fuel is provided by alcohol-based prep solution, drapes, sponges, endotracheal tubes. In the presence of a high oxygen environment, all of these substances can burst into flames and burn intensely.[1]

When alcohol-based prep is used and the patient draped before the solution is completely dry, alcohol vapors can be trapped and channeled to the surgical site or solution wick into the surrounding linen, where a heat source can ignite the vapors.[1] Experimental studies have shown that hot wire cautery or diathermy generates enough heat to ignite all alcohol-based antiseptics even if these contain as little as 20% alcohol. The ignition temperatures for these fluids are within the range of 800–900°C. These temperatures are easily reached with the use of typical electrocautery units.[2] Common cause for ignition of fire is use of electrocautery for hemostasis, before the evaporation of alcohol.[3] Till now, patient was burned during cardiac surgery,[3] and on the face, neck, and shoulders by the fire, which started during cranial burr-hole placement under monitored anesthesia care.[4] Severity of burns to patients is usually grade I, but it can be severe requiring plastic surgery.[1,3,4]

When alcohol-based skin preparations have to be used, a few recommendations have to be made which includes: a strict wait for 3 minutes for the solution to dry and the skin is wiped with a cotton swab before draping the surgical site;[1] shaving the skin especially in hirsute patients to prevent pooling of solution in the hair; effectively drape the patient with a clear plastic adhesive drape to prevent collection of flammable vapors beneath the drapes; when the patient’s trachea is not intubated (mask anesthesia) oxygen delivery should be kept to the minimum required to keep the oxygen saturation of blood within an acceptable range.[4]

The Hunter Health survey confirmed the risk of alcohol-based skin preparations and suggested strategies to reduce the risk of fire, including discontinuation of use of alcohol-based skin antiseptics in operating theatres; using fire retardant surgical drapes; installing over-current protection devices on electrical equipment; minimizing flammable conditions by avoiding nitrous oxide and using the lowest required concentration of inspired oxygen; using non-flammable cuffed endotracheal tubes; and education and training of operating theatre personnel in fire hazards.[5]
